# Contactless and Vibration-Based Damage Detection in Rectangular Cement Beams Using Magnetoelastic Ribbon Sensors

**DOI:** 10.3390/s23125453

**Published:** 2023-06-09

**Authors:** Christos I. Tapeinos, Maria D. Kamitsou, Konstantinos G. Dassios, Dimitris Kouzoudis, Aggeliki Christogerou, Georgios Samourgkanidis

**Affiliations:** 1 Department of Chemical Engineering, University of Patras, 26504 Patras, Greece; 2 Department of Mechanical and Manufacturing Engineering, University of Cyprus, Nicosia 1678, Cyprus

**Keywords:** magnetoelastic materials, vibration sensors, concrete structures, Metglas ribbons, structural health monitoring

## Abstract

This study investigated the innovative use of magnetoelastic sensors to detect the formation of single cracks in cement beams under bending vibrations. The detection method involved monitoring changes in the bending mode spectrum when a crack was introduced. The sensors, functioning as strain sensors, were placed on the beams, and their signals were detected non-invasively using a nearby detection coil. The beams were simply supported, and mechanical impulse excitation was applied. The recorded spectra displayed three distinct peaks representing different bending modes. The sensitivity for crack detection was determined to be a 24% change in the sensing signal for every 1% decrease in beam volume due to the crack. Factors influencing the spectra were investigated, including pre-annealing of the sensors, which improved the detection signal. The choice of beam support material was also explored, revealing that steel yielded better results than wood. Overall, the experiments demonstrated that magnetoelastic sensors enabled the detection of small cracks and provided qualitative information about their location.

## 1. Introduction

Structural health monitoring (SHM) plays a crucial role in ensuring the safety and operational efficiency of various structures. Failure without prior indication can have severe consequences on human well-being and daily productivity. Typically, damage begins internally within the material as microscopic cracks due to fatigue, and over time, these cracks propagate, posing a risk to the structure’s integrity. Detecting such cracks at the micro-scale is challenging and often necessitates expensive and intrusive methods such as electron microscopy or ultrasound techniques, which involve removing a small part of the structure for inspection. However, several cost-effective and non-intrusive techniques have been developed that can detect cracks in situ at a sub-millimeter scale, enabling early detection and warning for effective structural health monitoring.

These structural health monitoring (SHM) techniques employ various technologies and principles, each with its own advantages and disadvantages. For instance, Zoughi and Kharkovsky [1] conducted a review on near-field microwave and millimeter-wave techniques. Meenakumari et al. [2] utilized an inductive transducer for crack detection on railroad tracks. Qian et al. [3] developed a microwave resonator sensor capable of not only detecting the presence of a crack but also determining its direction. A magnetic tunnel junction-based gradiometer was employed in [4] for crack detection in cement. Kumar Sah et al. [5] designed an optical displacement sensor using Fiber Bragg Gratings specifically for crack monitoring. Kharkovsky and Giri [6] devised a technique involving a three-axis scanning mechanism for crack detection using microwave imaging. Yang et al. [7] introduced a balanced-field electromagnetic technique for detecting cracks in pipelines. The Electro-Mechanical (E/M) impedance method, as described in [8], allows for direct identification of structural dynamics by analyzing its E/M impedance or admittance signatures, which are significantly influenced by the presence of cracks. Sohn et al. [9] present a fatigue crack detection technique utilizing nonlinear ultrasonic wave modulation. Qaderi et al. [10] conducted a study on the nonlinear vibration analysis of a rectangular composite plate with cracks. The plate was inclined at an angle and reinforced with graphene platelets (GPLs). The plate was supported by a viscoelastic foundation and subjected to parametric excitation.

Strain sensors are highly significant in crack detection, as highlighted in several studies [11,12,13,14,15,16,17], due to their ease of installation on structures and cost-effective operation. These sensors typically consist of thin films and are minimally invasive. Damage-sensing approaches for cement-based materials evolved from conventional techniques, such as strain gauges (1950s), cement-based sensors (1990s), optical fibers [18] and piezoceramic sensors [19], to non-destructive methods (X-ray, C-scan and camera) [20,21] and lately to piezoresistive nano-sensors (carbon nanotubes and graphene) [22], piezoelectric strain gauges (ESG) [23], magnetic sensors [24] and wireless admittance [25]. Moreover, in the context of crack detection on beams, a limited number of techniques focus on capturing the bending spectrum of the beam [8,26,27,28,29,30,31,32,33,34,35,36], observing how the positions of the nodes change with the presence of cracks. In this study, magnetoelastic sensors are employed to measure strain, enabling the recording of up to three bending modes of cement beams and evaluating their performance when a single crack is introduced. Magnetoelastic materials, which are magnetic counterparts to piezoelectric materials, exhibit the ability to alter their magnetic state in response to mechanical stimulus, such as applied strain, and vice versa. A specific category of materials referred to as “Metglas” is characterized by their metallic amorphous nature and demonstrates a high conversion ratio between elastic energy and magnetic energy, known as the “coupling coefficient” (k). Metglas materials exhibit remarkable coupling coefficients, with values as high as 0.92 [37], surpassing those of other materials such as Nickel (0.31) or Fe-Co alloys (0.35). This exceptional characteristic makes Metglas materials highly suitable for sensing applications. Typically, Metglas materials consist of thin metallic amorphous ribbons, approximately 25 μm in thickness, and have demonstrated their potential in various sensing applications [38,39,40,41,42,43,44,45]. They offer several advantages, including low cost, the absence of a need for sophisticated and expensive electronic systems for interrogation (operating at low frequencies in the kHz range), and a contactless nature that eliminates the requirement for electric connections, as their signal relies solely on magnetic properties. Previous studies conducted by our laboratory successfully utilized these sensors for crack detection in aluminum cantilever beams [46,47,48,49].

In this study, the application of sensors for crack detection in simply supported cement beams is investigated. While extending previous work from aluminum to cement beams might appear straightforward, working with cement as a material presents inherent challenges. These challenges include high damping, which reduces the strength of bending modes and introduces high levels of noise, as well as the brittleness of cement, which necessitates special supports and careful excitation. The novelty of this research lies in the use of Metglas ribbons, a first-time application for detecting vibration spectra and cracks in cement beams. The affordability of Metglas ribbons enables their placement at multiple points in actual constructions, allowing for easy and contactless detection through the use of a pick-up coil. The findings of this study naturally pave the way for two future research directions. Firstly, there is a plan to investigate beams reinforced with steel, which are commonly used in large-scale construction projects. This will provide valuable insights into the behavior of such reinforced beams and the applicability of the sensing method in real-world scenarios. Secondly, the researchers aim to explore the possibility of embedding the sensing material within the cement beams. This concept has already been demonstrated with plastic beams in a previous study [50], showing that positioning the sensor close to the surface results in only a minor reduction in the detected signal. The intention is to apply a similar approach to cement beams, utilizing Metglas ribbons that will serve both as a sensing material and as reinforcement. This integration of the sensing material within the beam structure offers advantages such as addressing potential corrosion issues of Metglas in harsh environments. Overall, these future plans aim to expand the scope of the research by exploring steel-reinforced beams and incorporating the sensing material directly into the cement beams, thereby enhancing the effectiveness and practicality of the proposed sensing technique. Hence, our current work serves as a preliminary step towards achieving our long-term objectives.

The governing equation for the bending vibrations of a long beam can be represented by the following differential equation:

(1) $$\mathrm{EI}\frac{\partial^{4}w}{\partial z^{4}}+\rho A\frac{\partial^{2}w}{\partial t^{2}}=0$$

where the axis of the beam is assumed to be along the z-axis from z = 0 to z = L, w(z,t) is the vertical deflection of the beam (along x) out of the yz-plane, t is the time, A is the xy beam cross-section area, I is the second moment of area (an integral of the moment of inertia across A), E is the elastic modulus, and ρ is the density of the beam material. The problem is treated as one-dimensional along the beam axis, which is known as “long beam approximation” and is typically valid for $L>10\sqrt{A}$ . The usual separation of variables of z and t leads to vibrational solutions cosωt with respect to t and standing solutions with respect to z. For a simply supported beam with joint supports at its ends, the special boundary conditions are w = 0 and $\partial^{2}w/\partial z^{2}=0$ at both ends z = 0 and z = L, which leads to simple solutions of the form sin(k${}_{n}$ L) for the spatial part of w, with k${}_{n}$ = nπ/L, where n = 1, 2, 3,… The separation of variables leads to k${}^{4}$ = ρA$\omega^{2}$/EI, which results in bending mode frequencies of:

(2) $$f_{n}=\frac{\pi }{2L^{2}}\sqrt{\frac{\mathrm{EI}}{\rho A}}n^{2}$$

For a rectangular cross-section of area A = $\alpha^{2}$, the moment of area becomes:

(3) $$I=\frac{\alpha^{4}}{12}=\frac{A^{2}}{12}$$

Substitution of Equation (3) into Equation (2), results in:

(4) $$f_{n}=\frac{\pi \alpha }{4L^{2}}\sqrt{\frac{E}{3\rho }}n^{2}$$

## 2. Experimental Methods

### 2.1. Specimen Preparation

In this study, the bending modes of cement beams were measured and compared for the two cases of a crack-free beam and a beam with a single transverse crack on it. The beams used in the experiment were prepared entirely in proprietary laboratories using specific raw materials. These materials included:

(a) PlusCEM 52.5N commercial grey Portland cement from Durostick${}^{@}$ (Aspropyrgos, Attica, Greece) which is a type of CEM 52.5N cementitious hydraulic binder with reinforcing additives;
(b) Oil;
(c) Vaseline;
(d) Distilled water;
(e) Standard siliceous sand aggregate with a SiO${}_{2}$ content > 99%.

**Figure 1:**
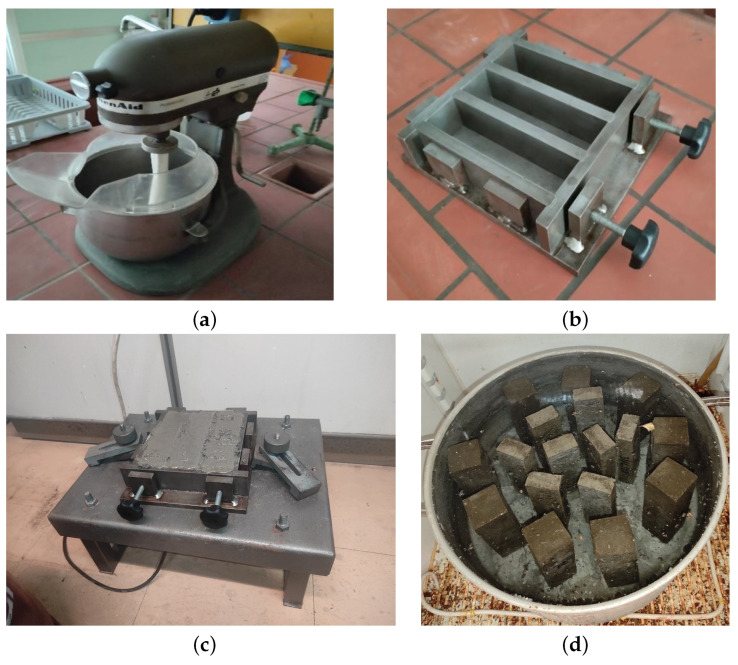
Tools used for specimen production: (**a**) a KitchenAid mixer (Whirlpool Corporation, Benton Harbor, MI, USA), (**b**,**c**) a mold with standard dimensions of 4 × 4 × 16 cm${}^{3}$ and (**d**) a container for keeping the cement beams in water according to EN 196-1 standard [51].

The weight ratio of water-to-cement-to-sand was maintained at 1:2:6. The cement specimens were prepared following the guidelines outlined in the EN 196-1 standard [51]. A professional mixer from KitchenAid (Figure 1a) and a mold with standard internal dimensions of 4 × 4 × 16 cm${}^{3}$ were used (as shown in Figure 1b,c). To facilitate demolding, the molds were lubricated with oil and Vaseline. After production, the filled molds were stored in a moist-air cabinet at a temperature of 20 ± 1 °C and 90% humidity for 24 h before demolding. Subsequently, the specimens were submerged in water, with proper spacing between each specimen (as shown in Figure 1d), and kept in the same cabinet at a temperature 20 ± 1 °C. The hydration process continued for 28 days before the testing phase commenced. As a result, the cement beams obtained from this procedure had dimensions of 4 × 4 × 16 cm${}^{3}$.

### 2.2. Experimental Setup

**Figure 2:**
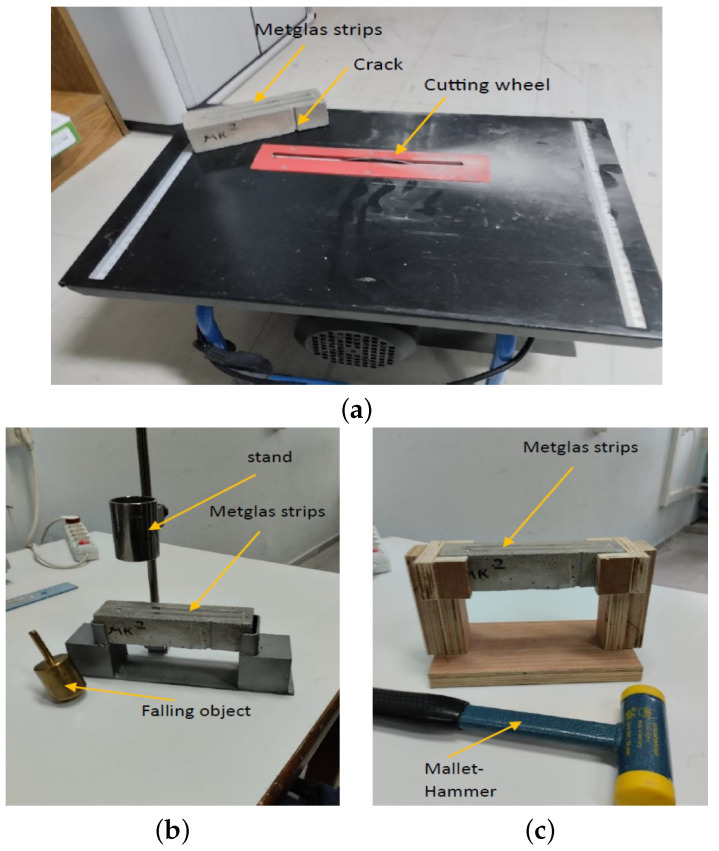
(**a**) A typical cement beam used with the sensors and the single crack on it plus the cutting wheel used to create the crack. (**b**,**c**) The experimental setup used for the excitation of the beam.

In Figure 2a, cement beams with dimensions of 4 × 4 × 16 cm${}^{3}$ were utilized in the experiments. Each beam was equipped with two magnetoelastic ribbons with dimensions of 13 cm × 7 mm × 25 μm, which were attached to the top face of the beam using thin double-sided tape.

These ribbons were composed of an alloy called “Metglas 2826MB3”, which is a common transformer core material manufactured by Metglas Inc. (Conway, SC, USA). The alloy’s average composition is provided in Table 1, while Table 2 presents the physical and magnetic properties of the alloy. To introduce a crack into the cement beam, a 4 mm cutting disk designed for ceramics was employed on a standard cutting-wheel table. The resulting surface crack exhibited a straight profile perpendicular to the beam axis with a width of 4 mm and an estimated depth of approximately 2 mm. After their fabrication, the specimens were placed on specially designed bracket supports (Figure 2b,c) to subject them to bending vibrations in a simply supported configuration. Two different brackets from two different materials were used in the experiments, namely a wooden bracket and a steel bracket. As shown in this figure, the excitation was different for each of the two brackets. In the case of the wooden bracket, a plastic hammer was used to excite the specimen, while for the steel bracket, a specially designed cylindrical stand was used to help the release of an 8 cm free-fall motion of a heavy 4.7 kg cone-like cylinder towards the specimen. An impact force of about 47 N was applied to the cement beam through the cone’s tip, which had a 3 mm diameter, leading to an impact stress of 6.6 MPa. The excitation in both cases was instantaneous and caused an initial bending displacement, after which the specimens were left free to vibrate. The method with the cone had better repeatability, as the release height was kept constant throughout all the experiments.

### 2.3. Signal Detection and Processing

**Figure 3:**
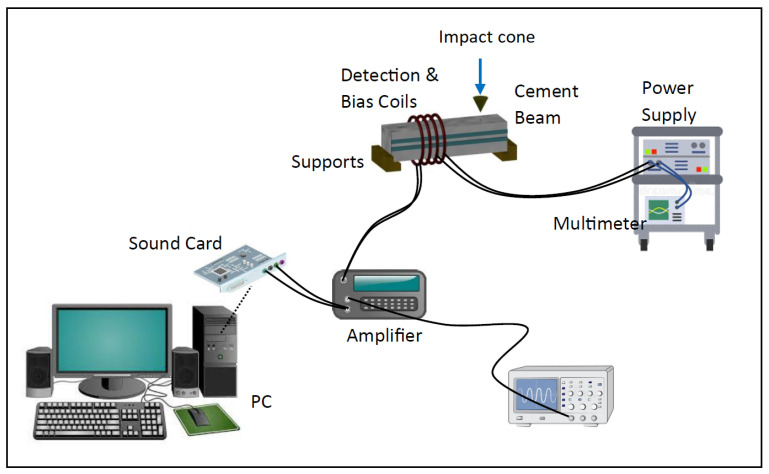
Schematic of the experimental setup used in the current study.

The sensing experiments were conducted using the experimental setup depicted in Figure 3. Two coils were wrapped around the cement beam in a contactless manner. As mentioned earlier, magnetoelastic materials have the unique ability to convert elastic energy to magnetic energy and vice versa, with maximum efficiency achieved when the material is subjected to a constant DC magnetic field. To achieve this, a DC current was applied to one of the coils using a power supply, thereby biasing the magnetoelastic ribbons. The other coil functioned as a detection coil responsible for detecting any changes in the magnetization of the ribbons. This detection signal was then transmitted to a PC sound card for data collection and transformation into a Fourier frequency spectrum. Additionally, the same signal could be observed on an oscilloscope.

**Figure 4:**
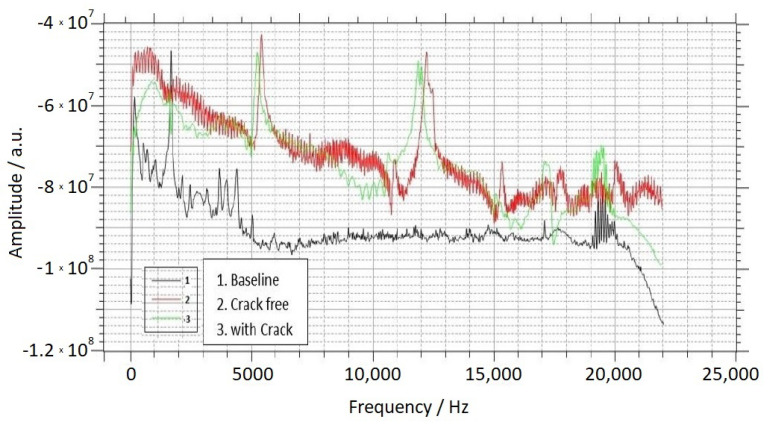
This Fourier spectrum displays the frequencies associated with the bending modes of the cement beam shown in Figure 2a. The three lines represent the measurements for the following scenarios: 1. without the presence of Metglas ribbons, 2. with Metglas ribbons attached to the beam but without any cracks and 3. with the addition of a crack in the beam.

Detection of the bending mode frequencies of the cement specimens was carried out using the following procedure. Since magnetoelastic materials such as Metglas can undergo changes in their magnetic state when subjected to mechanical deformation, alterations in the magnetic state of the Metglas ribbons can be directly linked to strain variations in the beam caused by the bending vibrations. The thinness of the Metglas ribbons (25 μm) ensures that they do not significantly impact the vibration characteristics of the beam, as previously demonstrated in our research [50] where we compared the elasticity of a beam with and without the attached ribbons. The detection coil, positioned in close proximity to the Metglas sensor ribbons, was capable of capturing their magnetization using Faraday’s law, which states that the voltage induced in the coil is proportional to the instantaneous strain of the beam. This voltage signal was acquired by a PC through an I/O card. The acquired voltage signal was subjected to Fast Fourier Transform (FFT) using specialized software called Audio Spectrum Analysis (Real-time Audio Spectrum Analyser 3.9.7.0). The resulting FFT transformation provided the bending mode spectrum of the beam, as illustrated in Figure 4. The three signals presented in the figure were obtained using the steel bracket and correspond to the following scenarios:

1. A cement beam without any cracks and without Metglas ribbons attached. This signal serves as a reference spectrum, primarily capturing surrounding electromagnetic noise (control spectrum).
2. A crack-free cement beam with attached Metglas ribbons.
3. The same cement beam as in Case 2, but with the presence of the side crack depicted in Figure 2a.

## 3. Results and Discussion

### 3.1. Main Idea

The spectra obtained, including the one shown in Figure 4, contain the essential information from the experiment, demonstrating (a) the capability to detect peaks corresponding to specific mode frequencies and (b) the ability to identify shifts in these peaks when a crack is introduced. In Figure 4, two prominent peaks are evident which are absent in the baseline signal, indicating that they correspond to the natural bending frequencies of the beam rather than noise from the coil. It is likely that additional peaks exist on the right side of the spectrum, but they are less distinct due to the significant damping observed in the cement beam compared to a metallic beam, which typically exhibits a greater number of discernible peaks [46]. The results indicate that the peaks shift towards lower frequencies when a crack is present.

To verify if the detected peaks correspond to bending modes, a numerical estimation is conducted using Equation (4). Although our beams do not satisfy the long beam condition due to their dimensions (cross-section area of 4 × 4 cm${}^{2}$ = 0.04 × 0.04 m${}^{2}$ and a length of 16 cm = 0.16 m), we can still check if the detected peak frequencies are within the same order of magnitude as those predicted by Equation (4). For the cement used in this study, a typical Young’s modulus value is E = 20 GPa = 20 × 10${}^{9}$ Pa, and the density value is ρ = 2300 kg/m${}^{3}$. Based on these values, the calculated fundamental resonance frequency from Equation (4) is f${}_{1}$ = 2090 Hz. However, as observed in Figure 4, such a peak is not present in the spectrum, but there are peaks around 5500 Hz instead. Although the two frequencies are in the same order of magnitude, they are not close.

The discrepancy between the predicted and observed frequencies can be explained by two possible reasons. Firstly, as was mentioned above, Equation (4) was derived assuming a thin beam model, where the thickness is typically less than one-tenth of the length. In our case, the beams have a square profile with a thickness equal to a quarter of the length, which does not satisfy the assumptions of the thin beam model. Therefore, the application of Equation (4) may not accurately predict the exact frequencies in our situation. Secondly, it is possible that the fundamental mode of vibration is not significantly excited, resulting in only higher modes appearing in the spectrum. This phenomenon was observed in our previous work [46], where the fundamental mode had either zero or minimal amplitude. It is plausible that similar behavior is observed in the current study. The underlying reason for this observation was not clear to the authors, but it was consistently observed when using thin and long metallic beams in previous experiments.

### 3.2. Results for Different Crack Locations

**Figure 5:**
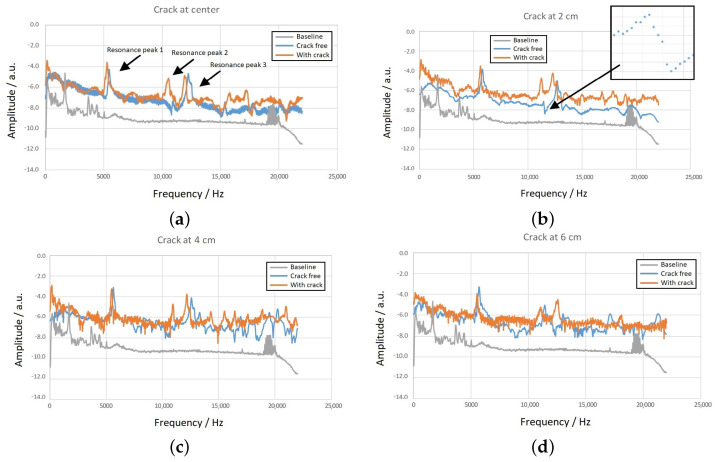
Shift of the bending mode spectra as a function of the crack location with respect to the beam center: (**a**) at 0 cm, (**b**) at 2 cm, (**c**) at 4 cm and (**d**) at 6 cm.

To investigate the impact of a single crack and its location on the spectrum, we introduced cracks at four different positions relative to the center of the beam. The cracks were placed at distances of (a) 0 cm, (b) 2 cm, (c) 4 cm or and (d) 6 cm from the center. Figure 5 displays the results for these four positions, where each graph represents a different crack location. Within each graph, the three signals shown are the same as those in Figure 4, the gray spectrum corresponds to the baseline without Metglas sensors, the blue spectrum represents the crack-free case, and the red line corresponds to the presence of a single crack at the specified position. Upon examining Figure 5, it becomes evident that there exists a frequency range of interest between approximately 5 kHz and 13 kHz, where three distinct peaks can be observed (labeled as 1, 2 and 3 in Figure 5a). Peaks 1 and 2 are present both before (blue line) and after (red line) the introduction of the crack. However, Peak 3 is more pronounced in the red spectrum, and initially, it does not seem to contribute significantly to the blue spectrum. Nevertheless, a closer examination of the inset in Figure 5b reveals that Peak 3 is indeed present, albeit small, and exhibits a resonance–antiresonance pattern.

**Figure 6:**
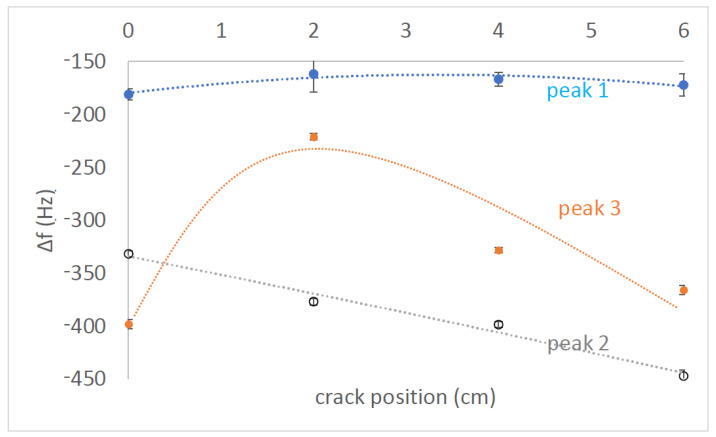
Frequency shifts Δf of the three peaks shown in Figure 4 with respect to the crack position.

Table 3 provides data on the crack-free frequencies (f${}_{\mathrm{free}}$ in Hz) for the three peaks, the corresponding frequency shifts (Δf ) after the crack introduction, and the absolute relative changes (Δf/f ) expressed as percentages (referred to as “RC” in the table). Each spectrum was recorded five times to ensure repeatability, and the values presented in the table represent the average of these five runs. Figure 6 graphically illustrates the variation of the data, with the points corresponding to the average values and the vertical bars indicating the standard deviation errors.

The significant feature of the frequency shifts is their magnitude, which allows them to be utilized as monitoring alarms for assessing the structural health of the beam. Furthermore, these shifts provide additional information. It is important to highlight that all Δf values in Figure 6 exhibit a negative trend, indicating that the introduction of a crack to the beam results in lower mode frequencies. The negative shift in mode frequencies can be explained by the theoretical expectation of Equation (1), as the introduction of a crack reduces the beam stiffness factor EI. On the other hand, the dependence of Δf on the crack location does not exhibit a consistent pattern across the three peaks. However, as previously demonstrated with metallic beams [46,47], this lack of a common tendency is advantageous for identifying the location of the peak. The relative changes in Δf for the four tested positions are provided in Table 4, illustrating the qualitative variations. There, the identifiers “small”, “medium” and “large” are used to represent Δf/f values of 2%, 3% and 4%, respectively.

It is observed that each crack position exhibits a unique “signature”, indicating that independent monitoring of the three peaks can provide information about the crack location. Previous successful implementations of this approach with metallic beams achieved crack position detection within 1% accuracy of the beam length and crack depth determination within 5% accuracy of the beam thickness [52]. However, it should be noted that cement beams generate less-distinct information in their vibration spectra compared to metallic beams, but it is anticipated that the same technique can be applied with reduced accuracy.

In terms of sensitivity, the relative volume change resulting from the introduction of the crack can serve as an indicator of the crack’s effect. With beam dimensions of 4 × 4 × 16 cm${}^{3}$, the total volume is 256 cm${}^{3}$. The crack dimensions in this case were a length equal to the beam’s width (4 cm), a depth of approximately 2 mm and a width of 4 mm, resulting in a crack volume of 0.32 cm${}^{3}$. The corresponding relative volume change, considering the decrease due to the crack, was calculated to be 0.125% (absolute value). Referring to Table 3, the relative frequency shifts were observed to be in the range of 2–4%. Taking the median value of these shifts as 3%, the sensing sensitivity or figure of merit can be evaluated as the ratio of 3% to 0.125%, resulting in:

(5) $$\mathrm{Sensitivity}=24\frac{\mathrm{Signal}\;\mathrm{change}\;(\%)}{\mathrm{Change}\;\mathrm{volume}\;(\%)}$$

### 3.3. Optimization

**Figure 7:**
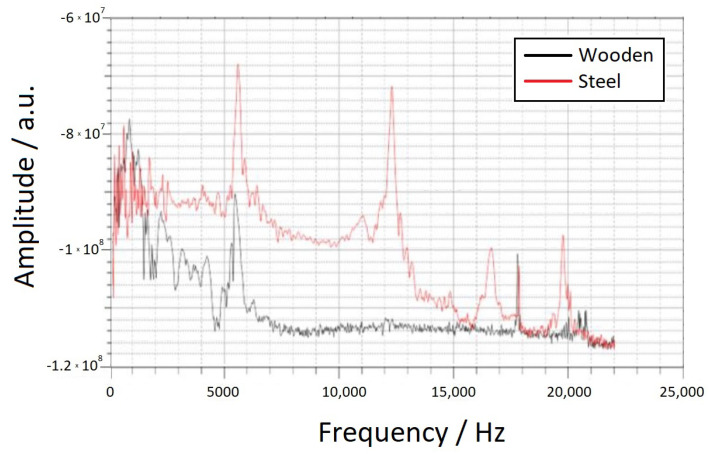
Spectra received by the same beam supported by two different supporting brackets made of different materials, where the black line corresponds to wood and the red line corresponds to steel.

In the experimental setup, two different materials, wood and steel, were used as supporting brackets to create the desired boundary conditions for the “singly supported beam” configuration. Figure 7 shows the spectra obtained from the cement beam when supported by these two brackets, with the wooden bracket represented by the black spectrum and the steel bracket by the red spectrum. From the figure, it is evident that while the peak on the left side is not significantly affected by the two materials, all the other peaks on the right side are attenuated in the case of the wooden bracket. This behavior can be attributed to the high damping characteristics of wood, which absorbs the high-frequency modes and reduces their amplitudes.

**Figure 8:**
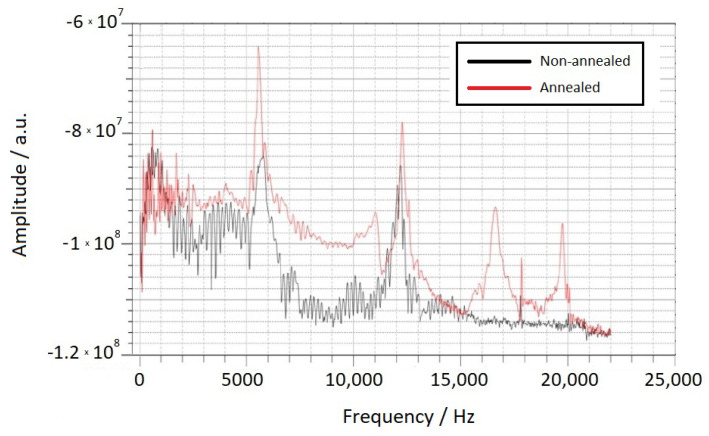
The spectra received by the same beam and the same sensor before (black line) and after (red line) thermal annealing.

Another factor that seems to influence the spectra is the thermal annealing of the Metglas sensors before their use. Thermal annealing helps to release internal stresses in the sensors, enhancing their ability to convert elastic energy into magnetic energy and improving their sensitivity [53]. For instance, Figure 8 illustrates the spectra obtained from the same beam and sensors before (black curve) and after (red curve) thermal annealing in an oven at 350 °C for 35 min. It is clearly noticeable that annealing significantly enhances the peak signal, amplifying its amplitude. This improvement can be attributed to the release of internal stresses in the Metglas material during annealing, allowing for better conversion of elastic energy to magnetic energy and thereby enhancing sensor sensitivity.

## 4. Conclusions

A novel sensing approach is proposed that involves placing magnetoelastic sensors on cement beams. This method aims to monitor the frequencies of bending modes and their changes when a crack is introduced at various positions on the beam’s surface. The experimental beams used in this study have dimensions of 4 × 4 × 16 cm${}^{3}$, while the cracks are 4 mm wide and 2 mm deep. The introduction of cracks results in relative frequency shifts ranging from 2% to 4%, which are significant enough to be used as early warning indicators for Structural Health Monitoring of the beam. The proposed method demonstrates a sensitivity of a 24% change in signal for each percentage change in volume when the crack is introduced. Although the frequency shifts exhibit non-linearity with respect to the crack location, they are mostly independent of each other, enabling their combination to provide accurate quantitative information about the crack’s position. The results reveal that using steel supports for simply supported beams produces better outcomes compared to wooden supports. Furthermore, subjecting the magnetoelastic sensors to thermal annealing at 350° for 35 min significantly improves the quality of the obtained signal from the sensors.

## Figures and Tables

**Table 1:** Metglas 2826MB3 elemental composition, data from material safety data sheet.

Element	Weight (%)
Boron	1–5
Iron	40–50
Molybdenum	5–10
Nickel	40–50
Cobalt (possible trace impurity)	0.3 (max)

**Table 2:** Metglas 2826MB3 physical and magnetic properties, data from Metglas Inc. website.

Physical Properties	Value	Magnetic Properties	Value
Density (g/cm${}^{3}$)	7.90	Saturation Induction (T)	0.88
Vicker’s Hardness	740	Maximum D.C. Permeability (μ)	-
Elastic Modulus (GPa)	100–110	Annealed	800,000
Tensile Strength (GPa)	1–2	As cast	>50,000
Lamination Factor (%)	>75	Saturation Magnetostriction (ppm)	12
Continuous service Temp. (°C)	125	Electrical Resistivity (μΩ cm)	138
Thermal Expansion (ppm/°C)	11.7	Curie Temperature (°C)	353
Crystallization Temperature (°C)	410	Anisotropy field (A/m)	300
Young’s modulus (GPa)	200	-	-

**Table 3:** Changes of the three peaks at the different crack positions. The quantities shown are crack-free frequencies f${}_{\mathrm{free}}$, their corresponding shifts Δf and the absolute relative change (RC) Δf/f expressed in %.

Crack Location	0 cm			2 cm			4 cm			6 cm		
Quantity	f${}_{\mathrm{free}}$	Δf	RC	f${}_{\mathrm{free}}$	Δf	RC	f${}_{\mathrm{free}}$	Δf	RC	f${}_{\mathrm{free}}$	Δf	RC
Units	Hz		%	Hz		%	Hz		%	Hz		%
Peak 1	5270	−190	4	5620	−170	3	5490	−150	3	5550	−150	3
Peak 2	11,930	−320	3	12,300	−410	3	12,170	−410	3	12,580	−450	4
Peak 2	10,550	−430	4	11,220	−220	2	11,070	−240	2	11,090	−340	3

**Table 4:** Qualitative relative changes to the data shown in Table 3.

Crack Location	0 cm	2 cm	4 cm	6 cm
Peak 1	Large	Medium	Small	Small
Peak 2	Small	Medium	Medium	Large
Peak 2	Large	Small	Small	Medium

## Data Availability

Data available on request from the authors.
